# Timely empirical antibiotic therapy against sepsis in a rural Norwegian ambulance service: a prospective cohort study

**DOI:** 10.1186/s12913-024-11827-x

**Published:** 2024-10-31

**Authors:** Lars-Jøran Andersson, Gunnar Skov Simonsen, Erik Solligård, Knut Fredriksen

**Affiliations:** 1 Anaesthesia and Critical Care Research Group, Faculty of Health Sciences, UiT - The Arctic University of Norway, Tromsø, Norway; 2 Division of Emergency Medical Services, University Hospital of North Norway, Tromsø, Norway; 3 Department of Microbiology and Infection Control, University Hospital of North Norway, Tromsø, Norway; 4 Research Group for Host-Microbe Interaction, Faculty of Health Sciences, UiT – The Arctic University of Norway, Tromsø, Norway; 5 Department of Research, Innovation, Education and Health Service Development, Møre og Romsdal Hospital Trust, Ålesund, Norway; 6 Department of Circulation and Medical Imaging, Gemini Centre for Sepsis Research, Norwegian University of Science and Technology, Trondheim, Norway

**Keywords:** Emergency Medical Services, Sepsis, Anti-Bacterial Agents, Ambulance, Emergency Medical Technicians, Paramedics, General practitioners, Primary Care Physicians

## Abstract

**Background:**

Early diagnosis and antibiotic therapy in patients with sepsis reduce morbidity and mortality, thus pre-hospital management is likely to affect patient outcomes. Pre-hospital administration may increase the risk of unnecessary use of broad-spectrum antibiotics, but identification of an infectious focus enables more targeted antibiotic therapy. The aim of this study was to investigate how paramedics, with or without the assistance of general practitioners, can administer empiric intravenous antibiotic treatment against sepsis in a timely manner.

**Methods:**

Cohort study of patients with suspected sepsis that received pre-hospital intravenous antibiotics and were transported to hospital. The setting was mainly rural with long average distance to hospital. Patients received targeted antibiotic treatment after an assessment based on clinical work-up supported by scoring systems. Patients were prospectively included from May 2018 to August 2022. Results are presented as median or absolute values, and chi-square tests were used to compare categorical data.

**Results:**

We included 328 patients. Median age was 76 years (IQR 64, 83) and 48.5% of patients were female. 30-days all-cause mortality was 10.4%. In cases where a suspected infectious focus was determined, the hospital discharge papers confirmed the pre-hospital diagnosis focus in 195 cases (79.3%). The presence of a general practitioner during the pre-hospital assessment increased the rate of correctly identified infectious focus from 72.6% to 86.1% (*p* = 0.009). Concordance between pre-hospital identification of a tentative focus and discharge diagnosis was highest for lower respiratory tract (*p* = 0.02) and urinary tract infections (*p* = 0.03). Antibiotic treatment was initiated 44 min (median) after arrival of ambulance, and median transportation time to hospital was 69 min. Antibiotic therapy was started 76 min (median) before arrival at hospital.

**Conclusions:**

Pre-hospital identification of infectious focus in suspected sepsis was feasible, and collaboration with primary care physicians increased level of diagnostic accuracy. This allowed initiation of intravenous focus-directed antibiotics more than one hour before arrival in hospital in a rural setting. The effect of pre-hospital therapy on timing was much stronger than in previous studies from more urban areas.

**Supplementary Information:**

The online version contains supplementary material available at 10.1186/s12913-024-11827-x.

## Background

Sepsis is a life-threatening condition characterised by a dysregulated host response to infection leading to organ dysfunction [1]. The global burden of sepsis in 2017 included 48.9 mill. patients worldwide and 11.0 mill. deaths, thus accounting for nearly 20% of all deaths [2]. In Norway, 246 patients/100 000/year were admitted to hospital with diagnosed sepsis 2008–2021 [3], and the overall age-standardised 30-day mortality was 16.9% [4].

Early diagnosis and initiation of intravenous antibiotic therapy reduce both morbidity and mortality [5–8]. Ambulance services can recognise sepsis and initiate treatment early, thus the management of sepsis in the pre-hospital setting is likely to affect patient outcomes.

Pre-hospital management strategies are dependent on available resources and personnel, and the standard of emergency care varies both between and within countries [9]. Ambulance-based diagnosis, blood culture sampling and administration of intravenous antibiotics is implemented in some services [10–13]. Still, high quality pre-hospital management of sepsis depends on several other elements like finding the correct focus of the infection and the timeliness of antibiotic treatment in this setting.

Using only one broad-spectrum antibiotic for sepsis in the ambulance simplifies the pre-hospital handling, as it eliminates both the need to identify an infectious focus and to select the appropriate drug. Consequently, it requires less training of staff and less drugs that must be carried by the ambulance. On the other hand, a system that includes different antibiotic regimens allows more targeted therapy aiming at an assumed focus of the infection and reduces unnecessary use of broad-spectrum antibiotics that have undesirable ecological effects.

However, a prerequisite for targeted antibiotic treatment is correct identification of the focus of infection in an acceptable fraction of the patients. To our knowledge, the ability of pre-hospital personnel to achieve this in patients with suspected sepsis has not been described earlier.

Likewise, timeliness of antibiotic administration is important, but time points and time intervals, like onset of sepsis and time to antibiotics, are not reported in a uniform way in the literature. Still, a clear description of the pre-hospital trajectory and time intervals may contribute to the understanding of what is the optimal timing of antibiotic administration in sepsis. Finally, we think that pre-hospital antibiotic treatment is particularly important in rural areas with long delays before Emergency Department (ED) admission.

The aim of this study was to investigate how paramedics, with or without the assistance of general practitioners, can administer empiric intravenous antibiotic treatment against sepsis in a timely manner.

## Materials and methods

### Study design

This was a cohort study with prehospital data collected prospectively and in-hospital data collected retrospectively.

### Inclusion and study period

Patients with suspected sepsis who were given pre-hospital intravenous antibiotics and transported to hospital by ambulance from May 2018 to August 2022 were included in this study.

### Study setting

The study was conducted in the catchment area of the University Hospital of North-Norway (UNN), that comprises the county of Troms and the Northern part of Nordland County in Norway. The area is large (30 412 km^2^) and sparsely populated with 193 066 inhabitants (01.01.2020) [14]. The population density is 6,3/km^2^, and a significant part of the population lives more than one hour from the closest hospital. There are hospitals in the cities of Harstad, Narvik and Tromsø. Ambulance personnel have two years of high school level training, plus a two-year apprenticeship in an ambulance service before final authorisation. Registered nurses can be authorised after two years of full-time ambulance work. In addition, an increasing, but still low, proportion of the personnel has a bachelor’s degree in paramedic sciences. Because of long distances, a significant proportion of ambulance patients are evaluated by general practitioners at primary emergency care centres before being transported to hospital.

### Local guidelines

Local guidelines for pre-hospital management of sepsis were developed in cooperation between the primary care services and the UNN Ambulance Department, and after publication of the Third International Consensus Definition [1] of Sepsis and the 2016 recommendations from the Surviving Sepsis Campaign (SSC) [15]. Antibiotic treatment was recommended if there was suspected sepsis after a clinical work-up that included scoring by systemic inflammatory response syndrome (SIRS) criteria and quick sequential organ failure assessment (qSOFA) score. The recommended antibiotics were gentamicin in combination with benzylpenicillin or ampicillin depending on suspected source of infection. Cefotaxime was a back-up alternative. It was recommended to give initial fluid resuscitation with crystalloids 10 ml/kg over 5–30 min, but the local guidelines also stated that as much as 30 ml/kg during the first 30 min might be necessary.

### Implementation of pre-hospital antibiotic treatment

The ambulance services implemented pre-hospital antibiotic treatment during May to August 2018. All ambulance personnel had e-learning and 4–6 h of lectures and practical training before they were certified to administer antibiotics. The training included recognition of usual signs and symptoms of sepsis, as well as use of SIRS and qSOFA. It was emphasised that the decision to give pre-hospital antibiotic treatment should be based on clinical presentation and that scoring systems should only be used for decision support. Attendees received practical training in blood culture sampling and preparation and administration of intravenous antibiotics. Blood culture sampling was identified as a particularly important and difficult procedure, and a step-by-step instruction video was produced to allow repetition after initial training. The ambulances were equipped with a sepsis kit for blood culture sampling (two vials for aerob sampling, two vials for anaerob sampling, and one vial for aerob sampling in patients ≤ 12 years) and four different types of antibiotics.

### The ambulance quality registry

After implementation of pre-hospital antibiotic treatment with antibiotics as a routine, a prospective quality registry was established. Data were collected by ambulance personnel with the online survey tool Research Electronic Data Capture (REDCap, Vanderbilt University Nashville, USA) immediately after treating patients with sepsis or suspected sepsis, and transferred to the ambulance quality registry. The inclusion criteria for the registry were suspected sepsis and either drawing of blood cultures or administration of intravenous fluids or intravenous antibiotics, or the perceived need for a sepsis team at arrival at the ED. Pre-hospital registration included administrative mission information, sepsis risk factors, vital signs, evaluation of skin, point-of-care laboratory results, suspected source of infection, triage system scoring, administration of antibiotics and fluids, cooperation with health personnel and adverse events. Pre-hospital data were supplemented by retrospective collection of in-hospital data from the electronic patient records at UNN. The in-hospital data included sepsis diagnosis at hospital arrival and discharge, treatment with antibiotics, fluids and vasoactive drugs after arrival at hospital, microbiological agents, criteria for septic shock, Charlson comorbidity score, sequential organ failure assessment (SOFA) -score, length of intensive care unit-stay, hospital stay and 30-day all-cause mortality (Supplementary materials 2 and 3).

### Selection of patients, blood culture sampling and antibiotic treatment

Treatment was initiated if sepsis was suspected based on clinical judgement supported by SIRS-criteria and qSOFA-score. If a primary emergency care physician was not present on the scene, ambulance personnel would consult a primary emergency care or hospital physician by telephone. Two sets of blood cultures consisting of two bottles each (one aerobic and one anaerobic) from the same venous puncture were drawn if possible, and antibiotics were given according to national guidelines (gentamicin + benzylpenicillin, gentamicin + ampicillin, or cefotaxime). Patients with a suspected lower respiratory tract focus of infection should receive gentamicin + benzylpenicillin, and patients with a suspected urinary tract focus should receive gentamicin + ampicillin. The local guidelines for patients with an unclear focus of infection was changed from gentamicin + ampicillin to gentamicin + benzylpenicillin in November 2019. Patients allergic to penicillin and children should receive cefotaxime. The goal was to give patients with suspected sepsis intravenous antibiotic treatment within one hour from first medical contact. If the assumed transportation time to hospital was less than 15 min, pre-hospital antibiotic treatment was not recommended. In the present study we included patients from the ambulance quality registry who received pre-hospital intravenous antibiotic treatment and were transported to hospital by ground or air ambulance.

### Statistical analyses

The data were pseudonymized and analysed with SPSS v.29.0.1.0 (IBM Corp. Armonk, USA). Results are presented as median with interquartile range (IQR), or as absolute values and percentages as appropriate. Chi-square tests were used to compare categorised data of source of infection and presence of general practitioner at initial assessment of patient. SankeyMATIC (Steve Bogart (https://githup.com/nowthis/sankeymatic)) was used to produce Sankey diagrams showing route for ambulance transports of patients admitted to hospital and change in assumed focus of infection between pre-hospital assessment and discharge papers. Differences with a *p*-value < 0.05 were considered significant.

## Results

**Fig. 1:**
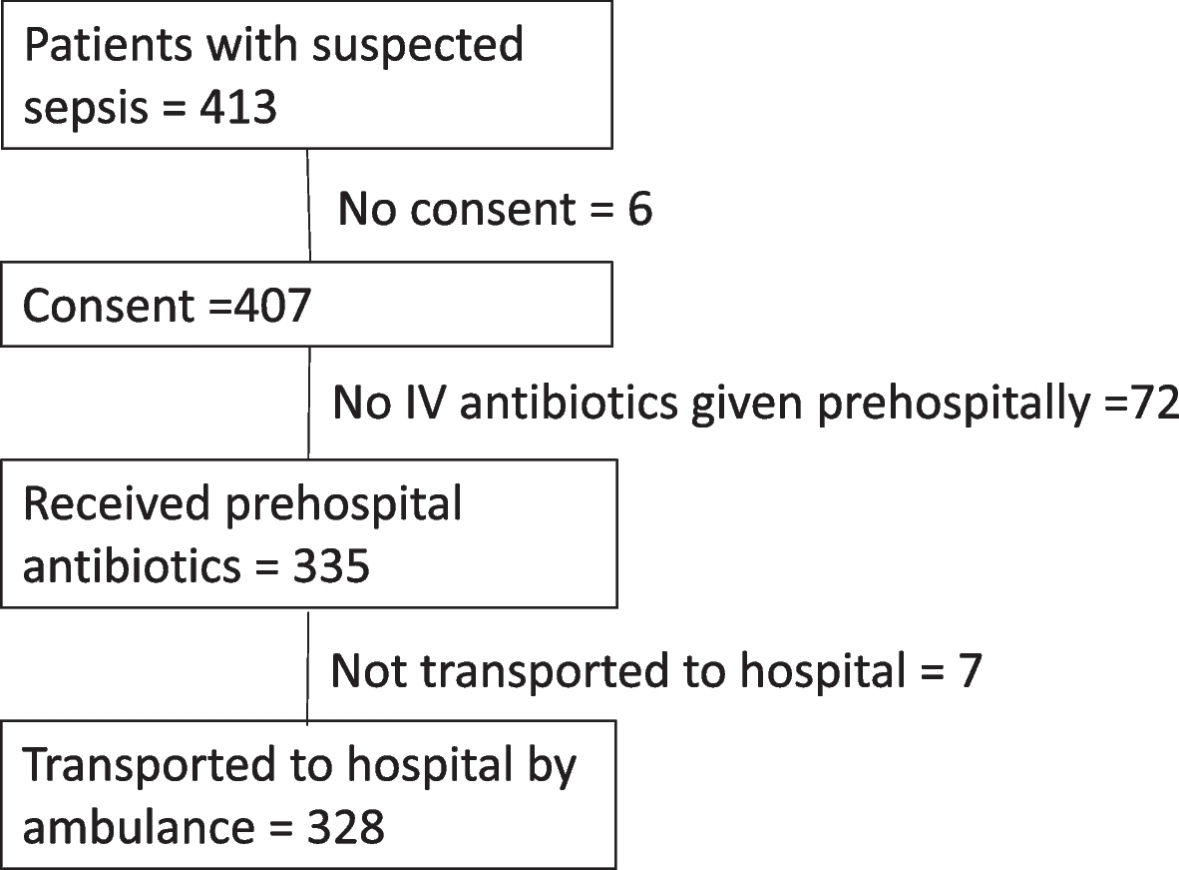
Inclusion of patients. 328 patients that were given antibiotics prehospitally and transported to hospital by ambulance were included

During the study period 413 episodes were recorded in the ambulance quality registry (Fig. 1). Six patients did not consent to participate in the study. Among the remaining patients, 335 received pre-hospital intravenous antibiotics. Seven were not transported by ambulance to hospital. These patients were nursing home residents, or had restrictions on level of care, and some were treated at municipal day care units. A table with demographic and clinical data of 71 patients who were transported to hospital, but did not receive prehospital antibiotics is available in supplementary material 1. Thus, the study group consisted of 328 patients corresponding to 42 cases of suspected sepsis per 100 000 inhabitants/year in the study period.

**Fig. 2:**
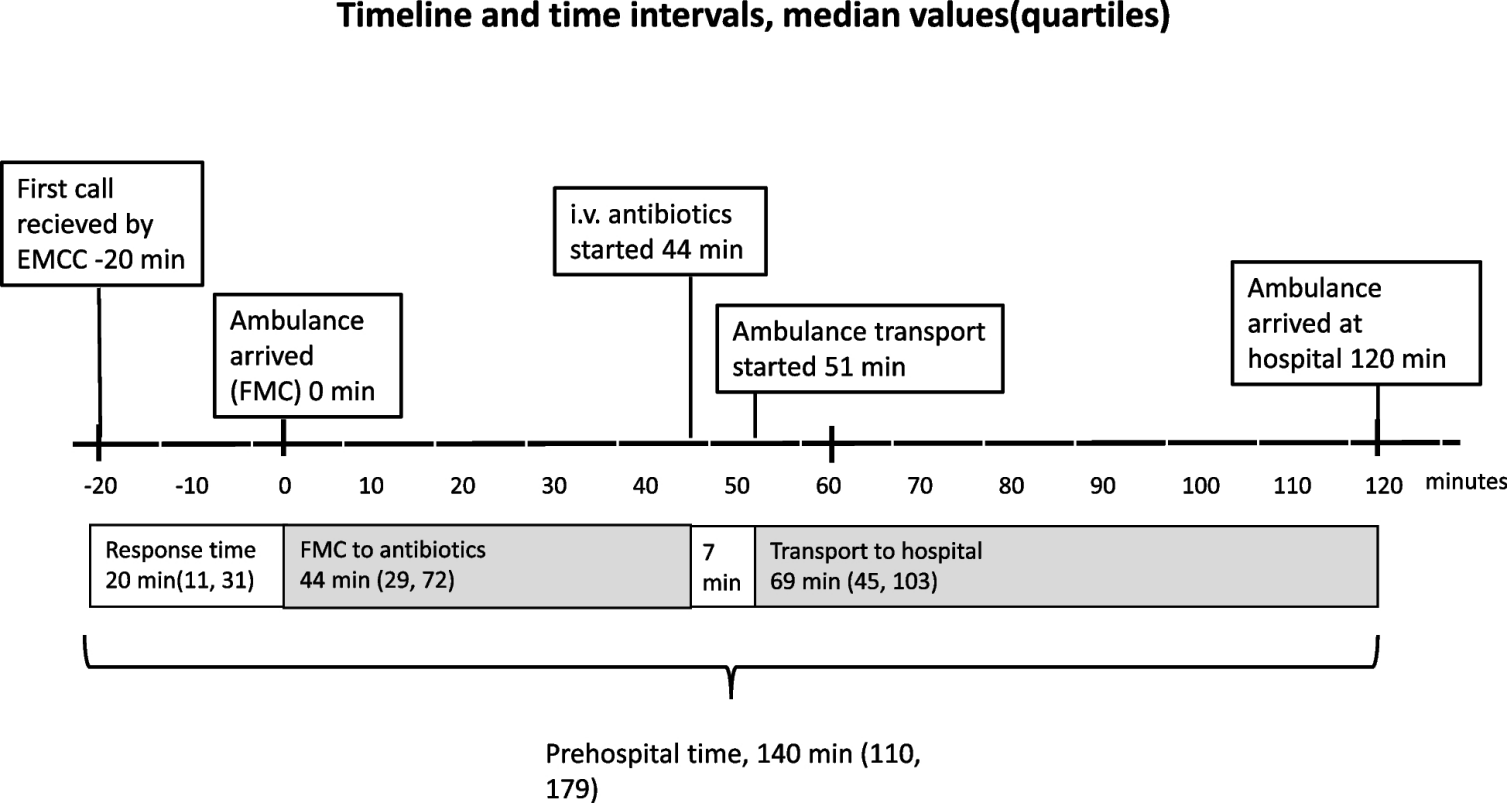
Timeline and time intervals. Time zero was defined as first medical contact (FMC, i.e. the time of arrival of the first medical personnel that could identify sepsis and start treatment with fluids and/or antibiotics). Response time is the time from the first call received by the Emergency Medical Communication Centre (EMCC) to FMC. Prehospital time is the time from first call received by the EMCC to ambulance arrival at hospital. The figures represent medians and interquartile ranges

Median time from first call received by the emergency medical communication centre to arrival of ambulance was 20 min (IQR 11,31) (Fig. 2). A primary care physician was present at start of treatment in almost half of the cases. The ambulances responded mainly to private residences (165 cases, 50.3%) or nursing homes (63 cases, 19.2%). The study group had a median qSOFA-score of 1 (IQR 1, 2), and the Charlson comorbidity score was 5 (IQR 3, 7). Median age was 76 years (IQR 64, 83) and 159 (48.5%) of the patients were female. 30-days all-cause mortality was 10.4% (*n* = 34). Demographic and clinical data from the patients are shown in Table 1.

**Table 1:** Demographic and clinical data. Continuous variables are presented as median values with interquartile range. Categorical variables are presented with number of patients and percentage

**Variable**	
Age (Years)	76 (64, 83)
Male (n)	169 (51.5%)
Female (n)	159 (48.5%)
Systolic BP (mmHg) (pre-hospital)	121 (104,141)
Temperature (Celsius) (pre-hospital)	38,5 (37.7, 39.0)
Respiratory rate (breaths/min) (pre-hospital)	28 (24, 30)
Heart rate (beats/min) (pre-hospital)	105 (92, 120)
Glascow Coma Score (pre-hospital)	15 (14, 15)
SpO2 (%) (pre-hospital)	93 (90, 95)
qSOFA-score (pre-hospital)	1 (1, 2)
Marbled or ashen skin (pre-hospital)	57 (17.4%)
Cyanotic skin, lip, or tongue (pre-hospital)	30 (9.1%)
SOFA-score after admittance to hospital	2(1, 3)
Charlson comorbidity score	5 (3, 7)
30-day all-cause mortality	34 (10.4%)
Primary care physician present in treatment	157 (47.9%)
Blood cultures drawn from the patient	298 (90.8%)

**Fig. 3:**
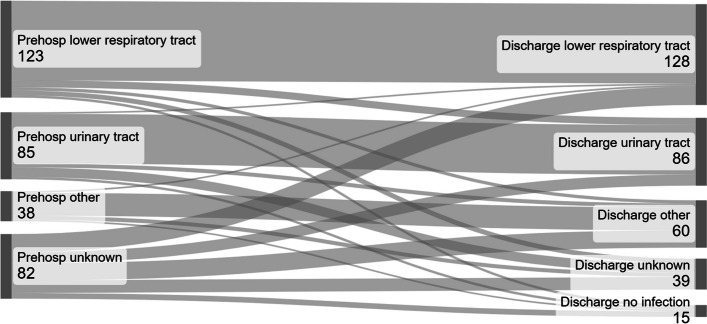
Infectious focus assumed by the pre-hospital personnel and according to discharge papers. To the left in the diagram, the focus of infection assumed by pre-hospital personnel, and to the right the focus that was concluded during the stay in hospital according to discharge papers. The figures are numbers of patients

At least one blood culture was drawn from 298 (90.8%) patients, resulting in 478 blood culture samples. Positive blood cultures were found in prehospital samples from 47 (15.8%) patients. In 123 (37.5%) cases the lower respiratory tract was the suspected source of infection, in 85 (25.9%) cases the urinary tract was the suspected source of infection, in 38 (11.6%) of cases other sources of infection were suspected, and in 82 (25.0%) of cases the source of infection was marked as unknown. In 246 cases (75%) there was a single suspected focus of infection in the pre-hospital setting. The number of cases with tentative single source of infection was not associated with the presence of a physician as it was documented in 77.7% of cases compared to 72.5% of cases without a physician present (*p* = 0.278). Discharge papers confirmed the pre-hospital tentative single source of infection in 195 cases (79.3%). The presence of a primary care physician led to a significantly higher rate of correctly identified source of infection 105/122 (86.1%) versus 90/124 (72.6%) (*p* = 0.009). In one out of four cases (*n* = 82) more than one focus was suspected as the source of infection, or the source of infection was classified as unclear (Fig. 3). There was a significantly higher proportion of cases with a discharge diagnosis of lower respiratory tract (*p* = 0.02) or urinary tract (*p* = 0.03) infections among patients with an identified pre-hospital tentative focus than among patients with an infection focus classified as unknown in the pre-hospital setting (Table 2).

**Table 2:** The focus of infection that was recognized at discharge from hospital in patients where one tentative focus was given in the pre-hospital setting, and in patients where source of infection was categorized as unknown or more than one source was suspected by the pre-hospital personnel

Focus of infection at discharge	Patients where a single tentative focus of infection was suggested by prehospital personnel (*n* = 246)	Patients where a single focus of infection was not suggested by prehospital personnel (*n* = 82)
Lower respiratory tract	105 (42.7%)	23 (28.0%)
Urinary tract	72 (29.2%)	14 (17.1%)
Other	38 (15.4%)	22 (26.8%)
Unknown	23 (9.3%)	16 (19.5%)
No infection	8 (3.3%)	7 (8.5%)

Antibiotic treatment was initiated at a median of 44 min (IQR 29,72) after arrival of ambulance, and 76 min (median) before arrival at hospital. Of the 328 patients who received pre-hospital antibiotics, 244 patients (74.4%) received therapy in accordance with national guidelines. Cefotaxime was administered in 66 cases (20.1%). Table 3 shows the combinations of antibiotics given in the pre-hospital setting.

**Table 3:** The table shows antibiotics given by the pre-hospital personnel, and whether the treatment is compliant with National guidelines for empirical antibiotic therapy for sepsis

**i.v. Antibiotic Treatment**	
Benzylpenicillin + Gentamicin (compliant with guidelines)	104 (31.7%)
Ampicillin + Gentamicin (compliant with guidelines)	84 (25.6%)
Cefotaxime (compliant with guidelines)	56 (17.1%)
Gentamicin	48 (14.6%)
Benzylpenicillin	17 (5.2%)
Cefotaxime + Gentamicin	8 (2.4%)
Ampicillin	7 (2.1%)
Benzylpenicillin + Ampicillin	2 (0.6%)
Gentamicin + Ampicillin + Cefotaxime	1 (0.3%)
Ampicillin + Cefotaxime	1 (0.3%)

**Fig. 4:**
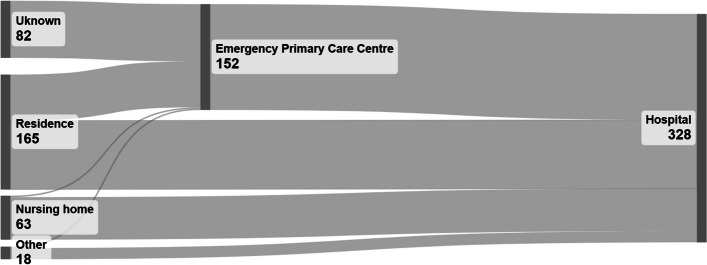
The figure shows the trajectories of the patients, from the location where the ambulance crews started the care and the route to hospital. “Unknown” are patients that the ambulance team first encountered at the Emergency Primary Care Centre where they had arrived by private transport

In most cases (176) patients were transported directly to hospital, including 61 patients who were nursing home residents (Fig. 4). In 70 of these cases patients were transported to the primary care centres by ambulance, only two patients were nursing home residents. Primary emergency care centres received 82 patients who arrived by other means of transportation. Altogether 152 patients were transported by ambulance from a primary emergency care centre to hospital. Median transportation time from place of incident to hospital was 69 min (IQR 45,103), and the median time from the first phone-call received by the emergency medical communication centre to arrival at hospital (total pre-hospital time) was thus 140 min (IQR 110,179).

The pre-hospital diagnosis of suspected sepsis was still assessed as likely at admission to hospital in 213 patients (64.9%), and intravenous antibiotics were continued for more than 36 h after admission in 286 patients (87.2%).

## Discussion

In this study we have shown that the introduction of intravenous antibiotic therapy for suspected sepsis was feasible in an ambulance service with long pre-hospital transportation times. The median time interval from arrival of ambulance to administration of intravenous antibiotics was less than one hour, which for most patients meant much earlier initiation of therapy than if antibiotics should be given after arrival in the ED. Blood cultures were drawn in 90% of the patients, and furthermore, pre-hospital personnel identified a tentative focus of infection which was confirmed after admission in 79.3% of cases. This allowed the pre-hospital team to start a targeted antibiotic therapy as opposed to broad-spectrum drugs. Only one adverse reaction (skin rubor after injection of benzyl penicillin) was reported.

The population studied was relatively old with comorbidities, and many of the patients were nursing home residents. This has also been found in other studies that report an average age as high as 70–75 years [10–12], and a high proportion of nursing home residents [12]. Mortality in the present study was 10.4%, which is in the same range as in a recently published randomised controlled trial (RCT) [10].

In recent studies, Walchok et al. [12] reported an average transportation time to ED of 16 min, whereas Cunningham et al. [13] reported a median scene plus transportation time to hospital of 39 min. Finally, Alam et al. [10] reported a median time of 26 min from pre-hospital administration of antibiotic treatment until arrival at ED. The latter study has been attributed much weight as it is the only RCT, and Alam et al. found no effect of pre-hospital antibiotics on major outcomes [10]. However, because of the short transportation times in this RCT, the findings may not be valid for rural areas with longer pre-hospital times. In the present study, antibiotics were given 44 min (median) after arrival of ambulance and 76 min (median) before arrival at the ED. In the trial conducted by Alam et al. antibiotics were administered 26 min (median) before arrival at the ED [10]. This is an important difference and illustrates how the potential benefit of getting earlier antibiotic treatment varies both between and within ambulance services. We believe that pre-hospital antibiotic therapy may have a greater impact in rural areas, like in the present study.

The much longer pre-hospital time in our study may be explained by the long distances in the catchment area. However, we may have some degree of selection bias, because antibiotic treatment was not offered if the hospital was closer than 15 min. Nevertheless, we report much earlier antibiotic administration in the patients we have studied than if therapy had been started after admission.

It is challenging to compare timelines with other reports, since authors use different terminologies and different starting points. In time-critical conditions both patient-delays and health-service system delays contribute to the total delay to treatment. In the case of community acquired sepsis, the onset of the disease is difficult to determine, particularly because of the insidious nature of the symptoms.

Both ED arrival time, and the time for recognition of sepsis have been used as time zero [16]. In our opinion, first medical contact is a well-established starting point for measuring intervals in time-critical medical conditions, like e.g. acute coronary syndrome [17]. Seymour et al. [18] pointed out that using time from ambulance arrival to start of therapy adds important and modifiable time intervals but did not define first medical contact unambiguously. We suggest defining first medical contact as when the first health professional arrives at the scene of incidence and can identify sepsis and start treatment with antibiotics and/or fluids. It is worth noting that this gives a much earlier starting point for the patient trajectory than studies that start the timeline in the ED and enables a timing of antibiotic treatment that is earlier in the development of the sepsis pathophysiology, which again may be important for patient outcome. However, more recent guidelines assume that the optimal timing of antibiotic therapy in sepsis depends on the probability of a bacterial infection and the severity of disease. In the present study, we have not differentiated between suspected sepsis, probable sepsis, or septic shock, as suggested by the 2021 Surviving Sepsis Campaign Guidelines. We only report that the assumed pre-hospital diagnosis was confirmed after ED admission in a high proportion of patients. Further studies should address how pre-hospital personnel may stratify patients according to individual needs for timing of therapy.

Internationally, many emergency medical systems operate without involving a doctor. We report that a primary care physician was present in almost half of the cases when treatment was started, and 28.4% of the patients were initially transported to a primary emergency care centre and this might have prolonged the pre-hospital time. On the other hand, close collaboration between local physicians and ambulance personnel might have contributed to better care decisions. Primary care physicians have an active role in pre-hospital emergency care in Norway, as every local community always has a general practitioner on call, and this implies that a doctor is more often part of the pre-hospital team in Norway compared to many other countries. It is possible that some patients that were considered eligible for pre-hospital sepsis management by ambulance personnel, were deemed not eligible by the doctor. These patients were not included in the present study unless blood cultures were drawn, or treatment started before they were re-evaluated.

In three out of four cases the pre-hospital team suspected a specific tentative focus of the infection. In this subgroup of patients, the focus was more likely to be the lower respiratory tract or the urinary tract than in the group of patients where the focus of infection was classified as unknown. This may indicate that lower respiratory tract and urinary tract infections give more typical symptoms and findings that are easier to interpret than other conditions. The proportion of cases with a defined pre-hospital tentative focus was not associated with the presence of a primary care physician. For patients with a tentative focus of infection before admission, the focus was significantly more consistent with discharge papers if a primary care physician had been present when treatment was started. The physician may thus seem to add significant competence to identify the focus correctly.

Antibiotic treatment was given according to recommended combinations in three out of four cases. In the few studies of pre-hospital antibiotic treatment patients received antibiotic regimens based on assumed source of infection only in one study [12], whereas in the other studies pre-hospital antibiotic treatment has been limited to administration of a single broad-spectrum agent [11–13]. When more than one type of antibiotic treatment was given the antibiotics were not given simultaneously; if the ambulance was close to hospital they would start with gentamicin, and benzylpenicillin or ampicillin was given after arrival at hospital. If we take this into account, 292 pre-hospital antibiotic treatment combinations (89.0%) may be considered guideline compliant. Seventeen patients (5.2%) received benzylpenicillin in monotherapy. This is not in line with recommendations for treatment of sepsis in Norway [19], however, benzylpenicillin in monotherapy is recommended for community acquired pneumonia [20] and this may explain the monotherapy. Patients received cefotaxime in 66 cases (20.1%), even though this broad-spectrum cephalosporin is discouraged as a first-line choice in Norwegian guidelines. The fraction of broad-spectrum antibiotics given in the study group was close to the fraction of broad-spectrum antibiotics (21%) given in Norwegian hospitals in 2022 [21].

### Strengths and limitations

The study is based on prospective registration by ambulance personnel, and we therefore expect higher data quality compared to retrospective studies. Treatment of patients was done in close collaboration between primary care physicians and ambulance services, but the registration of data was done solely by ambulance personnel. This implies that patients that were treated at nursing homes and not transported to hospital might not have been included. It is also likely that some of the patients had a non-resuscitation order or limitations regarding treatment or level of care that could influence inclusion in the study, but this was not recorded. We have not checked all ambulance transports for sepsis patients as this would require screening about 30 000 ambulance patient records per year. The rate of under-triage and underreporting in this study is thus unknown.

## Conclusions

Empirical intravenous antibiotic therapy was feasible in the described setting, and adherence to national guidelines for antibiotic usage was adequate. The pre-hospital times were longer in the rural setting of the present study compared to other published studies, and therapy was thus initiated much earlier than if it had to wait until arrival at the ED. The close collaboration with primary care physicians introduced a higher level of diagnostic accuracy.

## Supplementary Information

Supplementary Material 1.

Supplementary Material 2.

Supplementary Material 3.

## Data Availability

The datasets generated during and analysed during the current study are publicly available from the Open Research Data depository of the UiT – The Arctic University of Norway.

## Abbreviations

ED: Emergency Department
UNN: University Hospital of North-Norway (UNN)
SSC: Surviving Sepsis Campaign
SIRS: Systemic Inflammatory Response Syndrome
qSOFA: Quick Sequential Organ Failure Assessment
SOFA: Sequential Organ Failure Assessment
IQR: Interquartile Range
