# Supplementary material for: Timely empirical antibiotic therapy against sepsis in a rural Norwegian ambulance service: a prospective cohort study
Source: BMC Health Serv Res. 2024 Oct 31;24:1320. doi: 10.1186/s12913-024-11827-x (PMC11526532; doi:10.1186/s12913-024-11827-x)
Supplement: Supplementary file 1 — Supplementary Material 1. [file 12913_2024_11827_MOESM1_ESM.docx]

| **Variable** | **Prehospital AB given (n=328)** | **No prehospital AB given (n=71)** |
| --- | --- | --- |
| Age (Years) | 76 (64, 83) | 78 (69, 84) |
| Male (n) | 169 (51.5 %) | 28 (39.4 %) |
| Female (n) | 159 (48.5 %) | 43 (60.6 %) |
| Systolic BP (mmHg) (pre-hospital) | 121 (104 ,141) | 124 (98, 142) |
| Temperature (Celsius) (pre-hospital) | 38,5 (37.7, 39.0) | 38.1 (37.1, 38.7) |
| Respiratory rate (breaths/min) (pre-hospital) | 28 (24, 30) | 26 (22, 30) |
| Heart rate (beats/min) (pre-hospital) | 105 (92, 120) | 100 (83, 120) |
| Glascow Coma Score (pre-hospital) | 15 (14, 15) | 15 (14, 15) |
| SpO2 (%) (pre-hospital) | 93 (90, 95) | 93.5 (90, 96) |
| qSOFA-score (pre-hospital) | 1 (1, 2) | 1 (1, 2) |
| Marbled or ashen skin (pre-hospital) | 57 (17.4 %) | 11 (15.5 %) |
| Cyanotic skin, lip, or tongue (pre-hospital) | 30 (9.1 %) | 5 (7.0 %) |
| SOFA-score after admittance to hospital | 2(1, 3) | 2 (2, 4) |
| Charlson comorbidity score | 5 (3, 7) | 5 (4, 7) |
| 30-day all-cause mortality | 34 (10.4 %) | 6 (8.5 %) |
| Primary care physician present in treatment | 157 (47.9%) | 25 (35.2 %)) |
| Blood cultures drawn from the patient | 298 (90.8%) | 56 (78.9 %) |

Comparison between patients included in the study and patients with suspected sepsis, who did not receive prehospital antibiotic treatment and were transported to hospital.
