# Supplementary material for: Timely empirical antibiotic therapy against sepsis in a rural Norwegian ambulance service: a prospective cohort study
Source: BMC Health Serv Res. 2024 Oct 31;24:1320. doi: 10.1186/s12913-024-11827-x (PMC11526532; doi:10.1186/s12913-024-11827-x)
Supplement: Supplementary file 2 — Supplementary Material 2. [file 12913_2024_11827_MOESM2_ESM.pdf]

The Ambulance Quality Registry, In-hospital Data

Date: / 20	Amisnr:
First contact EMDC:	

Diagnosis

□ Sepsis likely according to ER-records
□ Other diagnosisis more likely according to ER-records
□ Sepsis included as diagnose at discharge
□ Infection diagnosis at discharge
□ Organ failure diagnosis at discharge

Proposed source of infection

Source of Infection/	Prehospital	Emergency Department	Discharge	Verified
Urinary tract				
CNS				
Abdominal				
Lung				
Skin				
Postoperativ Wound Infection				
Other Source of Infection				
Unknown Source of Infection				

Antibiotics

Itravenous antibiotics was continued for the first 36 hours after hospital admittion □
Time with empirical treatment before change to speficic AB therapy:

Fluids and vasoactive medication

Volum of fluids given within the 3 first hours from arrival at hospital:
Intravenous fluids given behfore arriavel at hospital □
Intravenous fluids started after arrival at hospital □
Time from arrival at hospital to start of treatment with intravenous fluids:
Vasoactive treatment started within the first 24 hours from arrival at hospital □

Mikrobiology

Blodculture sample number:
Microbiological agent found in blood culture, agent:
Microbiological agent from other sources found, agent:
Contaminated blood culture probable, agent:

Characteristics of patients

Charlston comorbidity index score	
Lactate	
Criteria for septic shock present during the first 24 hours of admittance to hospital (Adecvatly rescucutited with fluids and in need of vasoactive medication to reach MAP>65 mmHg)	

Highest SOFA-score in the first 24 hours after admittion to hospital	
Respiration	(0-4)
CNS	(0-4)
Cardiovascular	(0-4)
Liver	(0-4)
Coagulation	(0-4)
Kidneys	(0-4)
SOFA-score:	(0-24)

Outcome

Patient alive 30 days after admitton to hospital □
Length of stay in ICU:
Length of stay in hospital:
