# Supplementary material for: Timely empirical antibiotic therapy against sepsis in a rural Norwegian ambulance service: a prospective cohort study
Source: BMC Health Serv Res. 2024 Oct 31;24:1320. doi: 10.1186/s12913-024-11827-x (PMC11526532; doi:10.1186/s12913-024-11827-x)
Supplement: Supplementary file 3 — Supplementary Material 3. [file 12913_2024_11827_MOESM3_ESM.docx]

# The Ambulance Quality Registry, Pre-hospital data

The form should be completed after all ambulance missions with suspected sepsis and:

| □ blood cultures drawn | □ intravenous fluids started |
| --- | --- |
| □ antibiotics is started | □ need for Medical Emergncy Admittance Team |

Mission information

| Date: / 20 | | | Ambulance unit: | | | | Amisnr: |
| --- | --- | --- | --- | --- | --- | --- | --- |
| Age: | | | | | Gender: | | |
| Municipality fo incidence: | | | | | | | |
| First contact with EMDC: | | | | | | | |
| Priority: | □ Immediate | □ Urgent | | □ Regular | |  |  |
| Dispatch criteria: | | | | | | | |
| Ambulnce arrived at scene of incidence: | | | | | | | |
| Ambulance transport started: | | | | | | | |
| Time for transfer of patient care: □ Emergency department □ Outpatient clinic □ Not transported by ambulance | | | | | | | |

sepsis risk factors

| □ Pregnant | | □ Nursing home resident | | □ age>75 year |
| --- | --- | --- | --- | --- |
| □ Age<1 year | | □ Recent surgery | | □ Wounds or damaged skin |
| □ Foreign body (Catheter, pacemaker, protesis) | | □ Weakened immune system due to illness or medicine | | □ Excessive alcohol consumption, drug abuse |
| □ Cancer | □ Diabetes | | □ Chronic organ failure: Kidneys. Lungs or liver | |

Vital signs

| GCS: | | Changed consiousness: | □ yes | □no |
| --- | --- | --- | --- | --- |
| Respiratory rate: | | Endtidal CO2: | | |
| Oxygen saturation: | | Supplemental oxygen: | | |
| Heart frequenzy: | | Blood pressure: / | | |
| Temperature: | | | | |
| Latest urine: | □ less than 12 hours | □12-18 hours | □more than 18 hours | |

skin

| □ Marbeled or ashen skin |
| --- |
| □ Cyanotic skin, lips tounge |
| □ Petechial rash |

Lab values

| CRP: | Platelets: |
| --- | --- |
| WBC: | Lactate: |

Suspected source of infection

| □ Urinary tract | □ Lower airways |
| --- | --- |
| □ CNS | □ Skin |
| □ Abdomen | □ Postoperative wound infection |
| □ Other site: | □ Unknown site |

Triage systems

| SIRS: __/4 | | | qSOFA: __/3 | | |
| --- | --- | --- | --- | --- | --- |
| RETTS: | □red | □orange | | □yellow | □green |

Blood culture

| □ 2 x aerob + anaerob blood culture drawn before start of antibiotics (time) | |
| --- | --- |
| Time Blood culture: | □ lack of equipment |
| Comments: | □ Difficult venous puncture |
|  | □ No time available |

Antibiotics

| Time antibiotics started: | | □ Benzylpenicilline | | □ Gentamicine |
| --- | --- | --- | --- | --- |
|  |  | □ Ampicilline | | □ Cefotaksime |
| Route of admistration | □ i.v. | | □ i.o. | □ i.m. |
| □ Antibiotics started before arrival of ambulance. Antbiotics not given by ambulance. | | □ Estimated transportation time less than 15 minutes, antibiotics not given | | □ Antibiotics not given, other reason |

fluids

| Time fluids started: | Ringeracetate: ml |
| --- | --- |
|  | NaCl 0,9%: ml |

Where was treatment started (Fluids and/or antibiotics)

| □ Private home | □ Legekontor/eller legevakt |
| --- | --- |
| □ Nursing home | □ Ambulance |

Present at start of treatment

| □ Physician (GP) | □ Ambulance |
| --- | --- |
| □ Legevaktssykepleier | □ Air Ambulance physician |

Physicician consulted

| □ Physician (GP) | □ Hospital specialist |
| --- | --- |
| □ Regular GP | □ HEMS-doc |

Adverse events

| □ Allergic reaction | □ Other adverse events |
| --- | --- |
